# The association between plasma zinc concentrations and markers of glucose metabolism in adults in Cameroon

**DOI:** 10.1017/S0007114523000223

**Published:** 2023-01-25

**Authors:** Camille M. Mba, Kerry S. Jones, Nita G. Forouhi, Fumiaki Imamura, Felix Assah, Jean Claude Mbanya, Nicholas J. Wareham

**Affiliations:** 1 MRC Epidemiology Unit, University of Cambridge School of Clinical Medicine, Cambridge, United Kingdom; 2 National Institute for Health Research Biomedical Research Centre Nutritional Biomarker Laboratory, University of Cambridge School of Clinical Medicine, Cambridge, United Kingdom; 3 Department of Public Health, Faculty of Medicine and Biomedical Sciences, University of Yaoundé 1, Yaoundé, Cameroon; 4 Department of Internal Medicine and Specialties, Faculty of Medicine and Biomedical Sciences, University of Yaoundé 1, Yaoundé, Cameroon

**Keywords:** plasma zinc, glycaemia, insulin resistance, Africa

## Abstract

An abnormal zinc status has been suggested to play a role in the pathogenesis of type 2 diabetes. However, epidemiological studies of the relationship between plasma zinc concentrations and diabetes are sparse and inconclusive. We aimed to investigate the association between plasma zinc concentrations and glycaemic markers (fasting glucose, 2-h glucose and HOMA-IR) in rural and urban Cameroon. We studied 596 healthy adults (63.3% women) aged 25-55 years in a population-based cross-sectional study. The mean plasma zinc concentration was 13.7±2.7 μmol/L overall, with higher levels in men (14.4±2.9 μmol/L) than in women (13.2±2.6 μmol/L), p-value < 0.0001. There was an inverse relationship between tertiles of plasma zinc and 2-h glucose concentrations (p-value for linear trend=0.002). The difference in 2-h glucose between those in the highest tertile of plasma zinc compared to the lowest was -0.63(95% CI -1.02 to -0.23) mmol/L. This remained significant after adjusting for age, sex, smoking status, alcohol intake, education level, area of residence, adiposity and objectively measured physical activity -0.43(-0.82 to -0.04). Similar inverse associations were observed between plasma zinc concentrations and fasting glucose and HOMA-IR when adjusted for socio-demographic and health-related behavioural characteristics. The current findings of an inverse association between plasma zinc concentrations and several markers of glucose homeostasis, together with growing evidence from intervention studies suggest a role for zinc in glucose metabolism. If supported by further evidence, strategies to improve zinc status in populations may provide a cheap public health prevention approach for diabetes.

## Introduction

The burden of diabetes has risen globally over the past three decades but at a faster rate in low and middle-income countries where 80% of people in the world with diabetes live ^(1)^. In Africa, an estimated 24 million adults had diabetes in 2021, and this has been projected to be 55 million by 2045. Over 70% of deaths in people with diabetes in Africa occur in those who are in an economically productive age group, which has substantial implications at the individual, household and societal levels ^(2)^. Therefore, identifying the determinants of this growing diabetes burden is a major public health concern. In sub-Saharan Africa (SSA), the rise in diabetes prevalence has been attributed to a shift in dietary patterns along with physical inactivity, driven in part by urbanisation ^(3)^. This dietary transition towards the consumption of processed foods is associated with diets that do not often meet recommended dietary intakes of some micronutrients ^(4)^.

Zinc is an essential trace element naturally found mainly in meat, poultry, dairy products, and seafood ^(5), (6)^. Fortified foods and plant foods like legumes and grains are also good dietary sources of zinc ^(7)^. Although there is evidence to suggest that zinc deficiency is a public health issue globally ^(8), (9)^, the limited set of studies from Africa, which are mainly in children and women of reproductive age, suggest that the magnitude of zinc deficiency in Africa may be greater than in other parts of the world ^(8), (9), (10)^. Based on estimates of dietary intake of zinc using national food balance sheets, 26% of people in Africa have inadequate zinc intake (compared with 16% globally) ^(9)^.

There is evidence that zinc plays a role in glucose metabolism ^(11)^. Findings from large prospective observational studies suggest an inverse association between dietary zinc intake and type 2 diabetes risk ^(12), (13), (14)^. However, these studies relied on self-reports to assess dietary zinc intake which is subject to measurement error and recall bias. Measurement of blood zinc concentration to assess zinc status provides an objective measure that complements data on dietary zinc intake ^(15)^. There are few studies on the association between blood zinc concentration and type 2 diabetes and the results of these are inconsistent. In a previous cross-sectional study in the US and a prospective study in Finland, plasma zinc was positively associated with diabetes prevalence or risk ^(16), (17)^. However, these studies were conducted in a context where the prevalence of zinc deficiency is low ^(9)^. In other studies in China where low plasma zinc concentration is more common, plasma zinc was either inversely associated with diabetes prevalence ^(18)^ or not markedly associated with diabetes ^(19)^. Thus, it is possible that observations of the association between plasma zinc concentrations and diabetes could be affected by the frequency of low plasma zinc concentrations in the population studied.

Despite the high prevalence of inadequate zinc intake in Africa compared with other regions of the world, we did not find any previous population-based study in an African population linking dietary zinc intake or zinc biomarkers to glycaemic markers. Dietary patterns in many low and middle-income countries are rich in intake of phytates, which bind to zinc and inhibit its absorption ^(15) (7)^. In such settings, blood zinc may be a better indicator of zinc exposure than dietary zinc intake. In a previous study in Cameroon, the prevalence of low plasma zinc concentrations (< 70 μg/dL) in women aged 15-49 years was 82% ^(20)^.

In this population-based study including participants from rural and urban settings of Cameroon, a country with a high prevalence of low zinc status ^(8), (9), (20)^, we aimed to examine the independent associations between plasma zinc concentration and glycaemic markers. We hypothesised that plasma zinc concentrations would be inversely associated with glycaemic markers given the high prevalence of low zinc concentration in Cameroon.

## Methods

### Study population and design

The methods used for this study have been described in detail elsewhere ^(21)^. In brief, this was a population-based cross-sectional study conducted in 2005-2006 in 2 urban sites and 2 rural sites in Cameroon. The urban sites were Yaoundé, the capital city of Cameroon (Centre region) and Bamenda, the capital city of the North-west region and the rural sites were Mbankomo in the Centre region and Bafut in the North-west region. All adults aged 25 to 55 years without a history of diabetes or cardiovascular disease were approached through door-to-door recruitment in the four sites. A total of 651 participants (rural: n = 303, mean age 38.5 ± 8.3 years; urban: n = 348, mean age 37.9 ± 9.1 years) agreed to take part in this study. The mean age and sex ratio of volunteers was similar to all 3854 eligible participants identified in the delimited areas. We excluded 55 participants who did not have blood samples available for plasma zinc analysis. Ethical approval was obtained from the Cameroon National Ethics Committee and all participants provided written informed consent.

### Data collection

Fasting and 2-h glucose post 75g oral glucose tolerance test were measured on fresh capillary whole blood using a Hemocue B-Glucose Analyzer (HemoCue AB, Ängelholm, Sweden) onsite. Fasting blood samples collected from all the participants were centrifuged at *~* 1400 *g,* and plasma aliquots were stored at -80°C. Plasma samples collected in were transported on dry ice by air to Cambridge, United Kingdom and stored at -80 °C until analysis.

#### Measurement of plasma zinc

Plasma zinc concentrations were determined by inductively coupled plasma mass spectrometry (ICP-MS) using a Perkin Elmer NEXION 300D at Southampton University Hospitals in 2022. An internal standard (rhodium) was added to the plasma diluted in 1 in 50 in distilled water and to the quality control to normalise for sample preparation and instrument variability. Samples were run against matrix-matched calibration solutions prepared with bovine serum (Sigma-Aldrich). The zinc isotope signals (^66^Zn) were compared against the internal standard to determine the concentration of plasma zinc. Quality control materials were run with each analytical batch and consisted of a certified reference material (Sero, Norway) and an in-house material. The inter-batch coefficient of variation was less than 10.1% and values for the CRM were within the acceptable range. External quality assurance was performed as part of the Trace Element Quality Assurance Scheme (TEQAS) (UK NEQAS, Guildford, Surrey, United Kingdom).

#### Measurement of metabolic markers

Fasting plasma insulin was measured by fluorometric assay on a 1235 AutoDELFIA automatic immunoassay system (kit by Perkin Elmer Life Sciences; Wallac Oy, Turku, Finland). C-reactive protein (CRP), plasma cholesterol and triglycerides were measured using automated assays on the Dade Behring Dimension RxL analyser. CRP was measured using a particle enhanced turbidimetric (PETIA) technique and total cholesterol, high density lipoprotein (HDL) cholesterol and triglycerides were measured by enzymatic method. LDL Low density lipoproteins (LDL) cholesterol concentrations were derived by the Friedewald formula (LDL = total cholesterol - (triglyceride / 2.2) – HDL), when triglyceride levels were < 4.5 mmol/L. These analyses were conducted at the National Institute for Health Research (NIHR) Cambridge Biomedical Research Centre (BRC), Core Biochemical Assay Laboratory.

#### Covariate measurement

Data on socio-demographic characteristics (age, sex, education level, rural or urban residence) and health-related behaviours (alcohol intake, smoking, physical activity, self-reported fruit and vegetable intake) were collected by interviewers using an adapted version of the WHO STEPS questionnaire ^(22)^. Based on responses to the questions “have you ever smoked any tobacco product/consumed a drink that contains alcohol?” and “do you currently smoke any tobacco product/did you consume a drink that contains alcohol within the past 12 months?”, smoking status and alcohol intake were categorised as never, past or current.

Self-reported and objectively measured physical activity data were collected in all participants. Data on self-reported physical activity was collected using the global physical activity questionnaire (GPAQ) and estimates of energy expenditure in different domains (work, leisure and travel) and overall physical activity energy expenditure (GPAQ PAEE) were derived in metabolic equivalents of task (METs)-min/week ^(22)^. Physical activity energy expenditure (PAEE) was measured objectively over seven continuous days using a combined heart rate and movement sensor (Actiheart; Cambridge Neurotechnology, Cambridge, U.K.). The validity of this method was assessed in this population against PAEE measured with doubly labelled water (*r*=0.40) ^(23)^. PAEE scaled for body weight was expressed as KJ/Kg/day after calibration using individual heart rate. Three categories based on time spent in minutes per day at different intensities of physical activity were created: < 1.5 METs, sedentary behaviour; 1.5–3 METs, light physical activity (LPA) > 3 METs, moderate to vigorous physical activity ^(24)^. Throughout this manuscript, we use the term PAEE to refer to objectively measured PAEE.

Three measurements of blood pressure were taken using an automated blood pressure measuring device (OMRON M4-1) on the dominant arm of the participants after at least 5 minutes of rest and at 1-minute intervals. The blood pressure value was computed as the average of the three recordings. Waist circumference was measured to the nearest 0.1 cm in participants wearing light clothing using a non-stretch fiberglass tape at the level of the midpoint between the lower costal margin and the anterior superior iliac crests and height measured using a standard rigid stadiometer. Body weight was measured using electronic scales and body composition assessed using bioelectrical impedance analysis (Tanita TBF-531 scales; Tanita UK, Uxbridge, Middlesex, United Kingdom). Body mass index (BMI, kg/m^2^) was computed as the body weight (kg) divided by the square of height (m^2^).

#### Outcomes

Outcomes were markers of glucose homeostasis including fasting glucose, 2-h glucose and homeostatic model assessment of insulin resistance (HOMA-IR)). HOMA-IR was computed using the formula = ([FPI × FBG]/22.5)), where FPI is fasting plasma insulin (mU/L) and FBG is fasting blood glucose (mmol/L) ^(25)^.

### Statistical analysis

All the statistical analyses were performed using Stata 15 (StataCorp, Texas, United States). Descriptive statistics are presented as means ± SD (or median and (25^th^-75^th^ percentile) for non-normally distributed data) or numbers and percentages. We tested differences in means using the t-test (or differences in medians using the Mann Whitney test) and differences in proportions using the chi-squared test. Using the sex-specific cut-offs for defining zinc deficiency recommended by the International Zinc Nutrition Consultative Group, we reported the proportion of participants with plasma zinc deficiency (< 10.7 μmol/L in women and < 11.3 μmol/L in men) ^(26)^. Linear trends across tertiles of plasma zinc were obtained from a linear regression model for continuous variables including tertiles of plasma zinc as a continuous exposure and chi-squared test for trend (Cochran-Armitage test or Cochran-Mantel-Haenszel test for categorical variables with 2 or ≥ 3 levels respectively). We fitted linear regression models adjusted for age and sex to identify potential correlates of plasma zinc.

To examine the independent associations between plasma zinc and glycaemic markers, we categorised plasma zinc into tertiles and fitted three statistical models incrementally adjusted for potential confounding variables. After fitting crude regression models, we further adjusted for age (continuous) and sex, and then for smoking (never, past or current), alcohol intake (never, past or current), level of education (less than primary school, completed primary school, secondary school and university), residential site (4 sites), PAEE (continuous) and BMI (continuous). P-values for trend were obtained from linear regression models including plasma zinc as an ordinal variable across tertile categories. HOMA-IR was log-transformed to account for its skewed distribution. Complete case analysis was performed.

In sensitivity analysis, a) with missing data (PAEE, n=53; 2-h glycaemia, n=9; HOMA-IR, n=5) assumed to be missing at random, we imputed missing data by using multiple imputation by chained equations to create 10 imputed datasets and using Rubin’s rules to combine estimates (27) ; b) we further adjusted for self-reported fruit and vegetable intake as a proxy for overall dietary quality in model 3; and c) replaced BMI by body fat in model 3. We investigated non-linear associations of plasma zinc with glycaemic markers by fitting restricted cubic splines with 3 knots corresponding to the 25^th^, 50^th^, and 75^th^ percentile of continuously distributed plasma zinc using model 3 and assessed the shape of the association with glycaemic markers. We tested for effect modification by sex, rural-urban residence and BMI categories on the association between plasma zinc and glycaemic markers and subgroup analysis was performed if the p-value for interaction was < 0.05.

## Results

The characteristics of the study participants are presented in Tables S1 and S2. Of the 596 participants with measurements for plasma zinc, 63.3% were women. The mean (± SD) age of the participants was 38.3 ± 8.6 years. There was no difference in the mean plasma zinc concentration between rural (13.5 ± 2.9 μmol/L) and urban (13.8 ± 2.6 μmol/L) participants, p-value = 0.35. The mean concentration of plasma zinc was 13.7 ± 2.7 μmol/L with higher levels in men (14.4 ± 2.9 μmol/L) than in women (13.2 ± 2.6 μmol/L), p-value <0.0001. Using pre-established cut-offs for plasma zinc deficiency, 13.8% of women had plasma zinc concentrations below 10.7 μmol/L and 11.9% of men below 11.3 μmol/L.

Level of education and smoking status were positively associated with plasma zinc while female sex, physical activity, self-reported vegetable intake, 2-h glucose and adiponectin were all inversely associated (Tables 1 and 2). There was no evidence of a linear trend in fasting glucose and HOMA-IR across increasing tertiles of plasma zinc.

After adjusting for age, and sex, positive correlates of plasma zinc were male sex (adjusted for age only) and measures of adiposity (Table S3). Measures of physical activity and self-reported intake of fruit and vegetables were negatively correlated with plasma zinc. Rural/urban residential site was not associated with plasma zinc concentrations.

Table 3 shows the results of multiple linear regression analyses between plasma zinc concentration and glycaemic markers. 2-hour glucose was lower by -0.63 (95 % CI -1.02 to - 0.23) mmol/L among those in the highest tertile of plasma zinc compared with those in the lowest tertile (p-value for linear trend 0.002) in unadjusted analysis. This remained significant in model 3 adjusted for age, sex, smoking status, alcohol intake, education level, area of residence, adiposity and objectively measured physical activity (β: -0.43(95 % CI - 0.82 to -0.04) mmol/L, p-value for linear trend = 0.03.

Similar inverse associations were observed between plasma zinc and fasting glucose and HOMA-IR after adjusting for potential confounders. Compared with participants in the lowest tertile of plasma zinc, being in the highest tertile was associated with lower fasting glucose (-0.25 (-0.48 to -0.01) mmol/L) and lower HOMA-IR (-0.23(-0.44 to -0.03)), both p-value for linear trend < 0.05 in a multivariable model adjusted for socio-demographic characteristics and health-related behaviours (model 3). Results were unchanged in sensitivity analyses further adjusting for self-reported fruit and vegetable intake or BMI replaced by body fat in model 3.

There was no evidence of a non-linear association between plasma zinc and any of the outcomes using restricted cubic splines. The test for interaction between sex, rural/urban area of residence or BMI categories and plasma zinc concentrations on any of the glycaemic markers was not significant.

## Discussion

In this population-based cross-sectional study of 596 participants in Cameroon, we observed that plasma zinc concentration was inversely associated with glycaemic markers (2-h glucose, fasting glucose and HOMA-IR). The inverse associations between plasma zinc and fasting glucose and HOMA-IR became significant only after adjusting for sociodemographic characteristics and health-related behaviours. To our knowledge, this is the first population-based study in a sub-Saharan African population to examine the relationship between plasma zinc concentrations and markers of glucose homeostasis.

There are limited data from representative surveys on plasma zinc distribution from Africa in part because of the financial and technical resources required to analyse plasma zinc compared with the assessment of dietary zinc intake. The mean plasma zinc concentration in our study was comparable to those reported in studies in the US ^(28)^ and Europe ^(29), (30)^, but higher than in previous studies in Africa (20), ^(31), (32), (33)^. This could be because the previous studies in Africa were conducted mostly in children (< 5 years) or women of reproductive age. These population sub-groups are known to have higher zinc turnover ^(29), (30)^. In addition, blood samples in some of the studies were collected in non-fasting participants and in the afternoons. Plasma zinc concentrations follow a diurnal variation and are higher in the mornings and in fasting participants ^(15)^.

Previous epidemiological studies on the association between dietary zinc intake and diabetes have been limited by measurement error of dietary zinc assessment and show inconclusive results ^(14), (34), (12), (13)^. The quantification of plasma zinc offers the advantage of being an objective marker of both dietary zinc intake and body stores but has not been widely applied to test diet-disease association probably owing to the high cost of the plasma zinc analysis compared with the assessment of dietary zinc intake using subjective methods. As a result, evidence from previous observational studies using plasma zinc concentration is limited, with the majority of the studies coming from China ^(16), (35), (36), (37) (38)^.

Our findings of an inverse association between plasma zinc concentrations and glycaemic markers are consistent with previous case control studies showing that higher plasma zinc concentration was associated with lower odds of type 2 diabetes ^(18), (36), (37)^. In contrast, In contrast, a cross-sectional study in the US in 5153 adults reported that higher serum zinc concentration was associated with higher odds of pre-diabetes and diabetes ^(16)^. A similar positive association between serum zinc and risk of type 2 diabetes was reported in a 20-year prospective study of middle-aged and older men in Finland ^(17)^. In these studies showing a positive association between serum zinc and type 2 diabetes, the prevalence of low blood zinc concentration was low and it is has been suggested that excessive bioavailability of zinc may lead to overactive β-cells and eventually β -cell failure due to prolonged overactivity of the β -cell ^(39)^.

Some of these previous studies investigating the association between plasma zinc concentration and diabetes prevalence or risk did not account for health-related behaviours such as physical activity that confound the relationship between zinc and glycaemia. In this study, self-reported fruit and vegetable intake and physical activity were inversely associated with plasma zinc concentrations. Fruit and vegetable intake may be a proxy for a diet low in meat or high in plant-rich diets in this study, which are high in phytates that bind to zinc to form an insoluble complex, thereby inhibiting zinc absorption in the intestines ^(7)^. The inverse association between plasma zinc concentrations and physical activity is consistent with previous studies suggesting that physical activity promotes higher zinc excretion in sweat and (34) urine.

Plasma zinc concentrations respond to zinc supplementation ^(31), (40)^. To date, published studies of supplementation trials of zinc for diabetes prevention and management are mostly of small sample sizes and short duration. Previous meta-analysis of intervention studies reported a reduction in fasting glucose, postprandial glucose, glycated haemoglobin, fasting insulin and HOMA-IR with zinc supplementation compared with controls ^(41), (42)^. Notably, over 80% of the studies included in this meta-analysis were of short duration (< 1 year) and from Asia, where inadequate zinc exposure from low dietary zinc and high phytate intakes are prevalent ^(9)^. A Mendelian randomisation study reported no causal association between blood zinc and risk of type 2 diabetes ^(43)^. However, uncertainty remains due to the small sample size and only two SNPs included in the analyses.

Mechanistic evidence of the potential role of zinc in the pathogenesis of type 2 diabetes comes from animal and human studies ^(11), (44), (45)^. Zinc has a positive effect on insulin signalling in the skeletal muscles by stimulating the tyrosine phosphorylation of insulin receptors, thus promoting glucose uptake ^(44)^. Moreover, zinc is found in abundance in the pancreatic β-cells and is essential for the synthesis of zinc-insulin crystals (insulin crystallisation), the form in which insulin is stored in the pancreas. It has been suggested that the type, size and morphology of the zinc-insulin crystals regulate the conversion of pro-insulin to insulin ^(45)^. Zinc also appears to be an insulin-mimetic with the potential to modulate insulin storage, secretion and receptor signal transduction ^(11), (44)^. Some of the anti-inflammatory effects of zinc could explain its beneficial role in diabetes. Finally, zinc also acts as a co-factor for superoxide dismutase and other enzymes against oxidative stress ^(46)^.

We did not find evidence of a non-linear association between plasma zinc and any of the glycaemic markers. However, a recent large cohort study in China showed a U-shaped relationship between dietary zinc intake and diabetes risk, with an inflection point at 9.1 mg/day ^(34)^. Future prospective studies are needed to confirm our findings of an inverse association between plasma zinc and diabetes. If evidence of a beneficial effect of zinc in diabetes is shown in intervention studies, public health strategies to increase dietary zinc intake may offer a cheap and complementary primary prevention approach for diabetes.

## Strengths and limitations

The major strength of this study lies in the use of an objective indicator of zinc status since measurement of plasma zinc concentration does not rely on memory. Inadequate zinc status may result from insufficient dietary zinc intake, but also poor dietary zinc absorption (e.g. high dietary phytate intake that inhibits zinc absorption.). Thus in low and middle-income settings where dietary phytate intake is high, plasma zinc may be a better indicator of zinc exposure than estimated dietary intake of zinc ^(10), (47)^. In addition, variability in the bioavailability of zinc from foods limits dietary zinc assessment using subjective methods ^(15)^. Food composition tables are sometimes unavailable for local food items to calculate zinc and phytate intakes accurately, which is a drawback as the zinc content of plant-based foods may be influenced by soil zinc concentrations and phytates inhibit dietary zinc absorption ^(15)^. Plasma zinc concentration has also been shown to respond to dietary zinc intake and zinc supplementation and is a useful indicator of zinc status at the population level ^(48)^. However, many factors independent of zinc status such as infections, inflammation, duration of fasting, pregnancy and hormonal contraceptives affect plasma zinc concentrations ^(26)^. We conducted these analyses in a population-based study with the inclusion of participants from rural and urban areas of Cameroon and adjusted for socio-demographic characteristics and health-related behaviours in the analysis.

This study has several limitations. The blood samples were not collected in trace element-free tubes and the resulting plasma zinc concentrations may have been affected by contamination from environmental zinc, including zinc from the tubes in which the samples were stored or even the long-term storage. However, plasma zinc appears to be relatively stable after long-term storage and the contamination from tubes has been shown to be minimal ^(49)^. Even if plasma zinc concentrations were affected by contamination or long-term storage, this is unlikely to affect our observed associations as the effect of storage or contamination was likely to be random. In this cross-sectional study, we used fasting glucose, 2-hour glucose and HOMA-IR as intermediate markers of key pathways to diabetes rather than simply the prevalence of diabetes. Fasting and 2-hour glucose are also the means by which diabetes diagnosis is made, thus we are able to study the links between zinc status and diabetes risk, plus study its relationship with insulin resistance as a major pathophysiological pathway to diabetes. The estimates of insulin resistance derived from HOMA-IR are strongly correlated with estimates from the hyperinsulinaemic clamp (r=0.88), widely considered the gold standard for assessing insulin sensitivity ^(25)^. The absence of a measure of insulin secretion meant we were unable to study how zinc status might on diabetes risk via an effect on early phase insulin secretion. Another limitation is the cross-sectional study design of this study, which means we cannot rule out the possibility of reverse causation as an explanation of the associations observed. For instance, it is possible that the chronic hyperglycaemia present in patients with diabetes increases oxidative stress by producing free radicals which leads to lower zinc concentrations since zinc has anti-oxidative properties. Diabetes may also lead to low levels of zinc due to the higher levels of zinc excretion from the kidneys ^(50)^. Despite our attempt to control for a wide range of potential confounders, our observed associations may be affected by residual confounding. Our study was conducted in healthy adults aged 25-55 years and our findings may not be generalizable outside of this age range and beyond the geographical location in which the study was conducted.

## Conclusion

Our population-based study in rural and urban Cameroon shows that plasma zinc concentration was independently inversely associated with fasting glucose, 2-h glucose and HOMA-IR. This suggests a role of zinc in glucose metabolism possibly involving both insulin secretion and insulin resistance. Given that zinc is a biomarker that is elevated by intake of protein-rich foods, further work is required to disentangle the specific effects of zinc on diabetes from the effects of the food groups that influence zinc status. Additionally, the current cross-sectional findings should be investigated in prospective study designs.

## Figures and Tables

**Table 1:** Socio-demographic and behavioural characteristics by tertiles of plasma zinc concentrations (Cameroon study, n=596)

Characteristics	Plasma zinc categorised by tertiles			p for linear trend
	1^st^	2^nd^	3^rd^	
Zinc (μmol/L), range	6.2 – 12.4	12.5 – 14.5	14.6 – 25.8	
Age (years)	38.7 ± 8.1	38.9 ± 8.9	37.3 ± 8.8	0.09
Sex, n(%)				
Women	146 (72.6)	127(64.1)	104(52.8)	<0.001
Education, n(%) (completed)				
< primary school	36(18.0)	37(18.7)	30(15.2)	
Primary school	100(50.0)	83(41.9)	81(41.1)	0.04
Secondary School	41(20.5)	53(26.8)	56(28.4)	
University	23(11.5)	25(12.6)	30(15.2)	
Smoking status, n(%)				
Never	165(82.1)	155(78.3)	144(73.1)	
Past	26(12.9)	26(13.1)	27(13.7)	0.02
Current smoker	10(5.0)	17(8.6)	26(13.2)	
Alcohol intake, n(%)				
Never	21(10.4)	17(8.6)	27(13.7)	
Past	14(7)	22(11.1)	22(11.2)	0.07
Current	166(82.6)	159(80.3)	148(75.1)	
Residence, n(%)				
Rural	100(49.7)	77(38.9)	98(49.7)	0.99
PAEE (KJ/Kg/day)	51.5 ± 23.6	51.3 ± 22.2	48.7 ± 23.7	0.26
Sedentary time (min/day)	960.9 ± 154.1	937.0 ± 148.8	968.9 ± 154.7	0.63
LPA time (min/day)	356.2± 103.7	381.2 ± 105.2	362.7 ± 108.8	0.57
MVPA time (min/day)	122.9 ± 90.3	121.7 ± 84.3	108.4 ± 85.3	0.13
GPAQ PAEE (KJ/Kg/day)	48.6 (6.7 - 146.9)	20.3 (3.3 – 113.7)	7.6 (3.2-71.2)	<0.001
GPAQ work (MET-min/week)	3840(0-12480)	0(0-10080)	0(0-5760)	<0.001
GPAQ leisure (MET-min/week)	0(0-0)	0(0-0)	0(0-0)	
GPAQ travel (MET - min/week)	1440(560-3360)	840(280-3360)	840(280-1680)	0.002
Fruit intake (times/week)	2(1-6)	3(1-5)	2(1-3)	0.09
Vegetable intake (times/week)	6(1-8)	4(2-8)	3(2-5)	<0.001

n=596 except for PAEE where n=543 Results are presented as arithmetic mean ± SD [or median (25th-75th percentile) for non-normally distributed variables] or n (%). p-values for trend are from a chi-squared test for trend for categorical variables or from a linear regression model for continuous variables including tertile of plasma zinc as a continuous exposure. PAEE, physical activity energy expenditure; LPA, light physical activity; MVPA, moderate to vigorous physical activity; GPAQ, Global Physical Activity Questionnaire; MET, Metabolic Equivalents of Task

**Table 2:** Metabolic characteristics of the population by tertiles of plasma zinc concentrations (Cameroon study, n=596)

Characteristics	Plasma zinc categorised by tertiles			p for linear trend
	1^st^	2^nd^	3^rd^	
Waist circumference^(cm)^	88.0 ± 12.2	89.2 ± 12.2	88.6 ± 12.0	0.63
BMI (kg/m^2^)	25.9 ± 5.3	26.4 ± 5.2	25.9 ± 5.2	0.97
Body fat (%)	28.8 ± 11.1	28.9 ± 10.7	26.7 ± 11.4	0.05
Systolic blood pressure (mmHg)	120.7 ± 20.5	122.6 ± 22.1	124.6 ±19.6	0.07
Diastolic blood pressure (mmHg)	75.2 ± 12.8	76.8 ± 13.9	77.3± 13.4	0.11
Fasting glucose (mmol/L)	4.82 ± 1.06	4.79 ± 1.31	4.71 ± 1.61	0.40
2-hour glucose (mmol/L)	6.68 ± 1.85	6.20 ± 1.78	5.99 ± 1.99	0.0003
Fasting insulin (pmol/L)	21.7(11.5 - 37.5)	18.8(11.0 - 34.8)	22.7(12.4 - 33.4)	0.63
HOMA-IR index	0.75(0.37-1.32)	0.66(0.34-1.23)	0.76(0.39-1.21)	0.48
Total cholesterol (mmol/L)	3.78 ± 0.97	3.85 ± 0.99	3.91 ± 0.97	0.18
HDL cholesterol (mmol/L)	1.20 ± 0.35	1.23 ± 0.34	1.24 ± 0.30	0.29
LDL cholesterol (mmol/L)	2.18 ± 0.84	2.25 ± 0.84	2.30 ± 0.84	0.17
Triglycerides (mmol/L)	0.75(0.61-0.93)	0.73(0.58-0.92)	0.74(0.57-0.98)	0.59
CRP (mg/L)	3.97(2.44-7.37)	5.28(2.69-8.39)	5.43(2.47-10.12)	0.44

(n= 596, except for 2-h glycaemia where, n=587 and HOMA-IR, n=591) Results are presented as arithmetic mean ± SD [or median (25th-75th percentile) for non-normally distributed variables] or n (%). p-values for trend are from a chi-squared test for trend for categorical variables or from a linear regression model for continuous variables including tertile of plasma zinc as a continuous exposure. BMI, body mass index; HOMA-IR, Homeostatic model assessment of insulin resistance; ; HDL, High density lipoprotein; LDL, Low density lipoprotein; CRP, C-reactive protein

**Table 3:** Associations between plasma zinc concentrations and glycaemic markers (Cameroon study)

	β-coefficient (95% confidence interval)			p-value for linear trend
	Tertile 1	Tertile 2	Tertile 3	
Fasting glucose (mmol/L) (n=543)				
Model 1	1.0 (ref)	-0.04(-0.29 to 0.21)	-0.20(-0.45 to 0.04)	0.10
Model 2	1.0 (ref)	-0.03(-0.27 to 0.20)	-0.18(-0.41 to 0.06)	0.14
Model 3	1.0 (ref)	-0.06(-0.29 to 0.17)	-0.25(-0.48 to - 0.01)	0.04
2-h glucose (mmol/L)				
(n=536)	1.0 (ref)	-0.49(-0.86 to -0.12)	-0.63(-1.02 to - 0.23)	0.002
Model 1	1.0 (ref)	-0.47(-0.83 to -0.11)		0.007
Model 2	1.0 (ref)	-0.42(-0.77 to -0.07)	-0.54(-0.94 to - 0.15)	0.03
Model 3			-0.43(-0.82 to - 0.04)	
HOMA-IR (n=540)				
Model 1	1.0 (ref)	-0.07(-0.27 to 0.14)	-0.02(-0.24 to 0.19)	0.81
Model 2	1.0 (ref)	-0.02(-0.22 to 0.18)	0.07(-0.14 to 0.28)	0.53
Model 3	1.0 (ref)	-0.13(-0.30 to 0.05)	-0.23(-0.44 to - 0.03)	0.02

HOMA-IR, Homeostatic model assessment of insulin resistance Model 1: Unadjusted Model 2: Adjusted for age and sex, Model 3: model 2 + smoking status, alcohol intake, education level, residential site (4 sites), body mass index (continuous) and objectively measured physical activity (continuous)
